# Reversible Photo-, Thermal-, and pH-Responsive Functionalized Wood with Fluorescence Emission

**DOI:** 10.3390/ma15031229

**Published:** 2022-02-07

**Authors:** Kaiwen Zheng, Jiakai Wu, Munan Huang, Farao Zhang, Junting Xu

**Affiliations:** 1 Department of Polymer Science and Engineering, Zhejiang University, Hangzhou 310027, China; 11429008@zju.edu.cn; 2 Hangzhou Caihe No. 2 Primary School, Hangzhou 310000, China; Lena_munan2018@163.com; 3 Ningbo MaterChem Technology Co., Ltd., Ningbo 315800, China; zfarao@materchem.com

**Keywords:** functional wood, photo-response, thermal-response, pH-response, fluorescence

## Abstract

A reversible photo-, thermal-, and pH-responsive high-performance functional wood with fluorescence has been prepared. The properties, structure, multi-response, fluorescence, water resistance, and corrosion resistance of original wood (ORW) and functional wood (FUW) were investigated with an X-ray photoelectron spectroscopy (XPS) spectrometer, a Fourier-transform infrared (FTIR) spectrometer, a N_2_ adsorption–desorption analyzer, an atomic force microscope (AFM), tensile tests, a scanning electron microscope (SEM), an ultraviolet–visible (UV–Vis) spectrophotometer, a fluorescence spectrometer, the equilibrium swelling ratio (ESR), and corrosion tests. The results of XPS, FTIR, N_2_ adsorption–desorption, and AFM exhibited that FUW was successfully prepared. Additionally, the results of the tensile test and SEM revealed that FUW had better mechanical properties than ORW, due to the filling of epoxy resin in the pores of the wood. Moreover, the UV–Vis and fluorescence spectra demonstrated that the introduction of epoxy resin induced multi-response and fluorescence functions to FUW. Furthermore, the data of ESR and corrosion test showed that the introduction of epoxy resin greatly improved the water and corrosion resistance of wood. This study provides ideas and methods for preparing novel high-performance multi-response FUW.

## 1. Introduction

Wood is widely used in construction, furniture, packaging, and other fields because of its advantageous properties such as renewable, light weight, high strength, and easy processing [1,2,3]. However, wood is generally used as a structural material, instead of functional material, leading to low added value [4,5,6]. If wood is functionalized, many new properties can be introduced on the basis of retaining the original advantages of wood, so as to significantly increase its added value, broaden its application field, reduce the felling of forest resources, and finally achieve sustainable development [7,8,9].

In the past few years, the study of functional wood has attracted people’s wide attentions. Li et al. [10] successfully assembled perovskite solar cells on transparent wood substrates. Its power conversion efficiency was up to 16.8%, which may pave the way for the integration of solar cells with light transmitting wood building structures for energy-saving purposes. Zhang et al. [11] prepared two kinds of transparent wood for energy-saving windows by dipping epoxy resin containing W/VO_2_ composite nanoparticles into delignified wood. These two kinds of W/VO_2_ transparent wood composites both exhibit outstanding thermoregulation ability when they are used as windows. Hai et al. [12] prepared all bio-based transparent wood by infiltrating cellulose nanofiber and chitosan suspensions into the bleached wood. The prepared transparent wood showed 80% total transmittance, 30–60% haze, good thermal stability up to 315 °C, and excellent UV shielding properties, suitable for solar cell application. Bisht et al. [13] prepared a functional transparent wood by the lignin modification bleaching of poplar wood veneers followed by infiltration with epoxy resin doped with an UV absorber (2-(2H-Benzotriazol-2-yl)-4, 6-di-tert-pentylphenol), which has rapid photo-discoloration, high photostability, and optical transmittance. Bi et al. [14] prepared fluorescent wood by dipping epoxy resin combined with carbon dots into delignified wood, which showed white light emission under ultraviolet light excitation and enhanced tensile strength. However, the existing preparation methods of functional wood are all realized by adding functional materials to the interior or surface of wood. The added functional materials have poor miscibility with wood, and may deteriorate the original properties of wood itself, such as mechanical and processing properties [15,16,17]. At the same time, most added functional materials are expensive and have certain toxicity, which will greatly increase the cost and may pollute to environment and cause harm to the human body [18,19,20].

To solve the above problems, we impregnated a new kind of epoxy resin into wood and prepared a new type of functional wood. This kind of epoxy resin had the advantages of multi-response, fluorescence, low cost, and strong mechanical properties, and water and corrosion resistance [21,22]. Therefore, the FUW prepared from this epoxy resin also had these advantages. Additionally, this FUW was “self-functional”; its multi-function was not achieved by adding external functional materials, but was achieved by changing the network structure of epoxy resin [21]. Thus, this FUW also had the advantages of low cost and good miscibility.

In this study, tris(oxiran-2-ylmethyl)amine and 4,4-diaminodiphenyl-methane (DDM) were heated until melting and fully mixed; then, the mixture was impregnated into delignified wood and cured to prepare the FUW. The compatibility, morphology, and structure of wood before and after functionalization were measured by XPS, FTIR, N_2_ adsorption–desorption test, AFM, and SEM. Tensile tests were used to measure and compare the mechanical properties of wood before and after functionalization. The color-change function and fluorescence of FUW were studied with UV–Vis and fluorescence spectrometers, respectively. The water and corrosion resistance of wood before and after functionalization was measured and compared with the ESR and corrosion tests. This study provides new ideas and methods for the preparation of multi-functional wood with low cost, high added value, high mechanical properties, and water and corrosion resistance, with prospective applications in intelligent windows, sensors, and other fields [23,24].

## 2. Experimental Section

### 2.1. Materials

Sodium hydroxide (NaOH), sodium sulfite (Na_2_SO_3_), sodium chlorite (NaClO_2_), acetic acid, ethanol, tris(oxiran-2-ylmethyl)amine, and 4,4-diaminodiphenyl-methane (DDM) were purchased from the Beijing Chemical Reagent Company in Beijing, China. All the chemical reagents used in this work were pure and analytical grade. Balsa wood and deionized water were supplied by us.

### 2.2. Preparation of Delignified Wood

The cut wood was soaked in alkali solution (40 g NaOH, 20 g Na_2_SO_3_, 500 mL H_2_O), deionized water, acid solution (10 g NaClO_2_, 6 mL acetic acid, 500 mL H_2_O), deionized water, and ethanol. The soaking lasted 12 h for each step. The soaking temperature in the first four steps was 80 °C, and room temperature in the final step. The solutions in each step were renewed every 4 h.

### 2.3. Preparation of FUW

**Figure 1:**
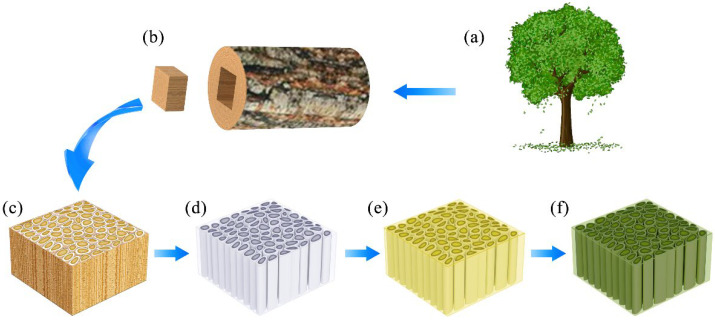
The preparation process of FUW: (**a**) tree; (**b**) wood; (**c**) ORW; (**d**) delignified wood; (**e**) FUW before the photochromic process; (**f**) FUW after the photochromic process.

First, tris(oxiran-2-ylmethyl)amine and DDM were weighed according to the molar ratio of epoxy group:active hydrogen = 1:1, heated to melt, and mixed uniformly. Subsequently, 10 g mixture was placed in a 60 °C vacuum oven to remove bubbles. Subsequently, the delignified wood was placed on the surface of the mixture for impregnation (0.04 MPa for 3 min, at atmospheric pressure for 1.5 min, and repeated the above steps 6 times). The dipped wood was then taken out of the mixed solution and placed in a mold to cure at 75 °C for 5 h, followed by curing at 105 °C for 5 h. Finally, the product (FUW) was taken out of the mold for characterization (Figure 1).

### 2.4. Characterization

X-ray photoelectron spectroscopy (XPS) of FUW was performed on an X-ray photoelectron spectrometer (ESCALAB 250, ThermoFisher Scientific Company, Waltham, MA, USA) with a monochromatic Al Ka source.

Fourier-transform infrared (FTIR) spectra of ORW and FUW powders were obtained on an FTIR spectrometer (Alpha, Bruker Company, Billerica, MA, USA) from 4000 to 400 cm^−1^, and the data were collected over 32 scans with a resolution of 2 cm^−1^ at room temperature.

N_2_ adsorption–desorption isotherms of ORW, FUW and delignified wood were obtained on a N_2_ adsorption–desorption analyzer (BK200C, JWGB SCI. & TECH. Company, Beijing, China) at −195.85 °C. The samples were degassed at 90 °C for 3 h before testing.

Atomic force microscopy (AFM) images of FUW were observed through an atomic force microscope (Nanoscope V Multimode, Bruker Company, Billerica, MA, USA) in tapping mode with a silicon cantilever (spring constant = 42 N/m, tip radius = 10 nm).

The stress and strain curves of ORW, FUW, and epoxy resin were measured on a universal testing machine (1185, Instron Company, Boston, MA, USA) at room temperature with a tensile rate of 10 mm/min. The size of the samples was 50 mm × 4 mm × 1 mm.

The morphologies of ORW and FUW were characterized through scanning electron microscopy (SEM) (S-4800, Hitachi Company, Tokyo, Japan). The surfaces of the samples were coated with gold before observation.

Simulation mechanical analyses of ORW, FUW, and epoxy resin were carried out with SolidWorks. The model of ORW was a hollow cube (155 mm × 155 mm × 85 mm) with many holes. The model of epoxy resin was a solid cube (155 mm × 155 mm × 85 mm) without holes. The model of FUW was also a cube (155 mm × 155 mm × 85 mm) but with many holes, although the holes were filled with epoxy resin. These models were fixed at one end and under a tensile stress of 400 N.

Ultraviolet−visible (UV−Vis) spectra of FUW film were obtained with an UV−Vis spectrophotometer (UV2550, Shimadzu Company, Kyoto, Japan) in the wavelength range of 200–800 nm.

Fluorescence spectra of FUW film were measured with a fluorescence spectrometer (F-7000, Hitachi Company, Tokyo, Japan). The slit values of the excitation wavelength (EX) and emission wavelength (EM) were 10 nm. At an excitation wavelength of 260 nm, fluorescence spectra with emission wavelengths from 280 nm to 800 nm were measured.

The water and corrosion resistance of ORW and FUW were studied by equilibrium swelling tests in water, acid solution (0.1 moL/L), alkali solution (0.1 moL/L), and salt solution (0.1 moL/L), respectively. The final value of the mass and volume of samples was an average value of five measurements.

## 3. Results and Discussion

### 3.1. Preparation and Compatibility of FUW

**Figure 2:**
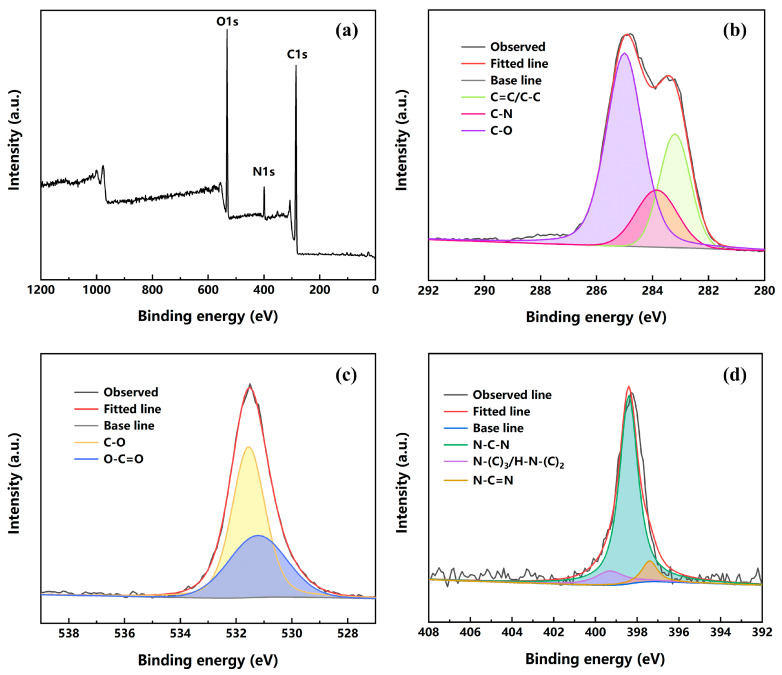
The XPS spectra of FUW (**a**); high-resolution XPS spectra of C1s (**b**), O1s (**c**), and N1s (**d**).

The chemical composition and type of groups of FUW were studied by XPS. Figure 2 exhibited the XPS spectrum of FUW, revealing the coexistence of carbon, oxygen, and nitrogen. Specifically speaking, FUW primarily comprised carbon (61.94 Atomic %), with a little oxygen (23.87 Atomic %) and some nitrogen (14.19 Atomic %) in it. The existence of nitrogen could be attributed to the dipping of epoxy resin. The high-resolution C 1s spectrum of FUW can be decomposed into three individual peaks, ascribed to C=C/C-C (283.19 eV), C-N (283.85 eV) and C-O (285 eV), respectively. Similarly, the O 1s spectrum of FUW can be resolved into two peaks at 531.56 eV and 531.21 eV, which are attributed to C-O and O-C=O, respectively. Additionally, there were three fitted peaks at 397.39 eV, 398.39 eV, and 399.29 eV in the N 1s spectrum of FUW, which corresponded to N-C=N, N-C-N, and N-(C)_3_/H-N-(C)_2_, respectively. These results revealed that FUW was successfully prepared.

**Figure 3:**
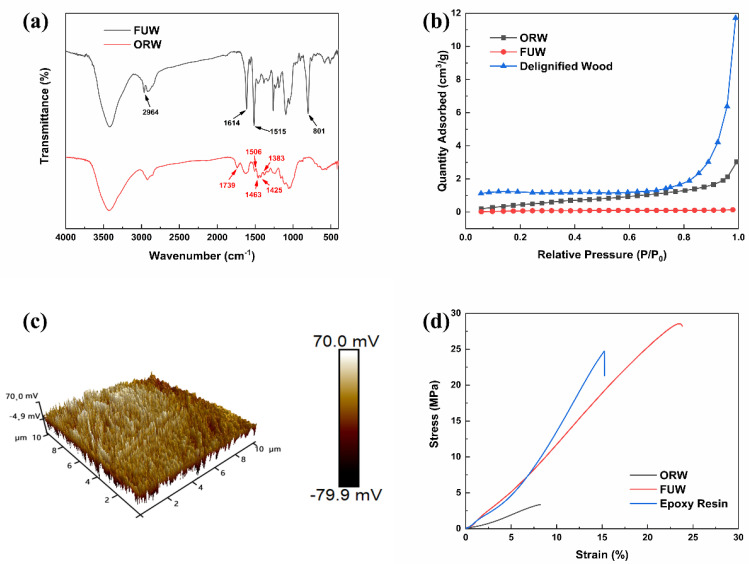
The FTIR spectra of ORW and FUW (**a**); N_2_ adsorption−desorption isotherms of ORW, FUW and delignified wood (**b**); AFM image of FUW (**c**); stress−strain curves of ORW, FUW and tris(oxiran-2-ylmethyl)amine/DDM epoxy resin (**d**).

The FTIR spectra of ORW, FUW, and epoxy resin are presented in Figure 3a and Figure S1. Apparently, the spectrum of ORW displayed peaks at 1383 cm^−1^ (C-H bending of cellulose), 1425 cm^−1^ (C−H aromatic in plane deformation of lignin), 1463 cm^−1^ (CH_2_ bending of hemicellulose), 1506 cm^−1^ (aromatic skeletal of lignin), and 1739 cm^−1^ (C=O stretching of lignin), which were the characteristic peaks of cellulose, lignin, and hemicellulose [25,26]. Moreover, the spectrum of FUW showed peaks at 801 cm^−^^1^ (C−H aromatic out-of-plane deformation), 1515 cm^−1^ and 1614 cm^−1^ (C=C aromatic stretching), and 2964 cm^−^^1^ (C−H of epoxy group), which were the characteristic peaks of tris(oxiran-2-ylmethyl)amine/DDM epoxy resin [27,28]. The results of FTIR confirmed that FUW was successfully prepared.

The specific surface area and pore properties of ORW, FUW, and delignified wood were assessed through N_2_ adsorption–desorption measurements (Figure 3b and Figures S2–S4). The curves of ORW and delignified wood showed a steep growth at high relative pressure (P/P_0_ > 0.8), revealing the existence of macropores. The results indicated that the specific surface area and macropores strongly increased after delignification, which meant that the delignified wood was successfully prepared. Additionally, the specific surface area became almost zero after dipping process, which meant that FUW was successfully prepared.

The AFM image (Figure 3c) showed the surface roughness of FUW. The low roughness indicated that the surface of FUW had a low crystallinity. This result revealed that epoxy resin had good compatibility with ORW, which strongly supported the conclusions obtained from XPS and FTIR.

### 3.2. Mechanical Property and Structure of ORW and FUW

Figure 3d shows the stress–strain curves of ORW, FUW, and epoxy resin. The tensile strengths of ORW, FUW, and epoxy resin were 3.36 MPa, 28.54 MPa, and 24.73 MPa, respectively. The elongations at break of ORW, FUW, and epoxy resin were 8.21%, 23.82%, and 15.27%, respectively. The results of tensile test showed that FUW had better mechanical properties than ORW and epoxy resin.

**Figure 4:**
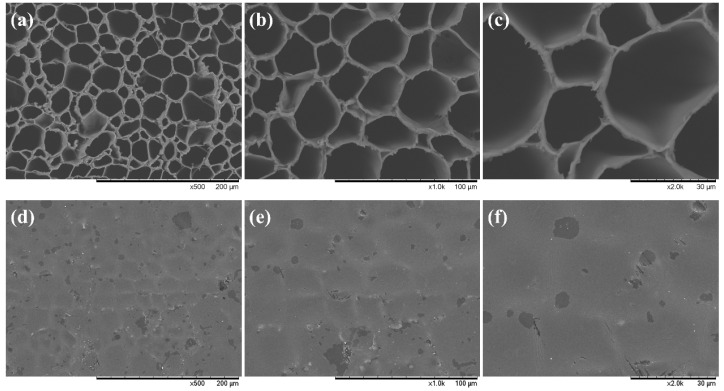
The SEM images of ORW (**a**–**c**) and FUW (**d**–**f**).

To further explore the reason for the excellent mechanical properties of FUW, we characterized the micro-structure of ORW and FUW with SEM. The obtained SEM images are displayed in Figure 4. The results showed that ORW had a honeycomb-like porous microstructure with a pore diameter of about 30 μm. This hollow structure gave wood advantageous light weight and high specific strength characteristics. In contrast, the microstructure of FUW was solid, the original hollow holes were filled with tris (oxiran-2-ylmethyl) amine/DDM epoxy resin, and the cellulose skeleton of wood was not destroyed but remained completely. Thus, epoxy resin micro-domains were introduced into the cellulose skeleton of wood. When micro-cracks were generated during the stretching process, the epoxy resin micro-domains could interrupt the further growth of cracks, thereby strengthening the wood phase. Under the circumstances, the mechanical properties of FUW were much higher than those of ORW. In turn, this structure can also be viewed as a three-dimensional and connected wood network introduced into the epoxy resin, in which the cellulose skeleton can toughen epoxy resin [29]. Therefore, the mechanical properties of FUW were even higher than those of pure epoxy resin. Finally, in this two-phase composite, the mutually reinforcing and toughening structure greatly improved the mechanical properties of FUW; thus, it can be applied to some specific fields where the mechanical properties of common wood are insufficient. Additionally, the densities of FUW, ORW, and epoxy resin are shown in Table S1. The density of wood strongly increased after functionalization, because the holes in the wood were filled with epoxy resin. This increase in density also leads to improvements in mechanical properties.

**Figure 5:**
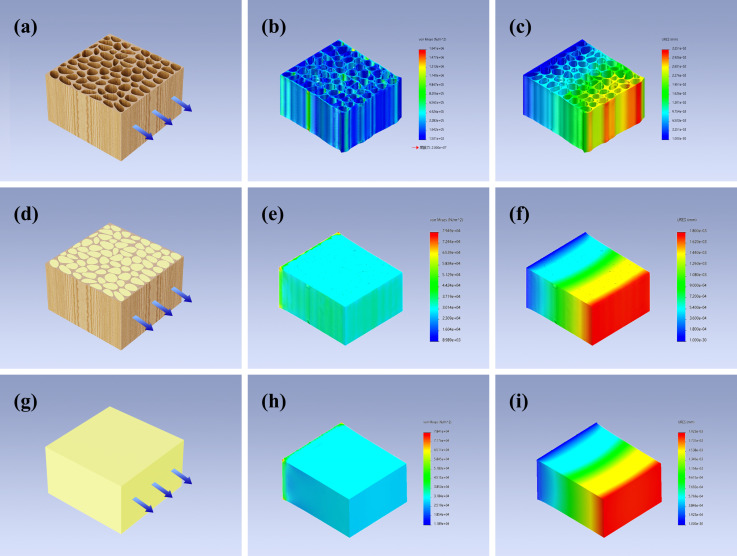
The abstract models of ORW (**a**), FUW (**d**), and epoxy resin (**g**); the stress nephograms of ORW (**b**), FUW (**e**), and epoxy resin (**h**); the displacement nephograms of ORW (**c**), FUW (**f**), and epoxy resin (**i**).

After clarifying the structure of ORW, FUW and tris(oxiran-2-ylmethyl)amine/DDM epoxy resin, their simulation mechanical analysis was studied by SolidWorks [30]. The abstract models, stress nephograms, and displacement nephograms of ORW, FUW, and epoxy resin are shown in Figure 5. The model of FUW had the minimum stress concentration and deformation, revealing that FUW had better mechanical properties than ORW and pure epoxy resin. The results of the simulation mechanical analysis strongly supported the conclusions obtained from the tensile test and SEM.

### 3.3. Photo-, Thermal-Response, and Fluorescence of FUW

**Figure 6:**
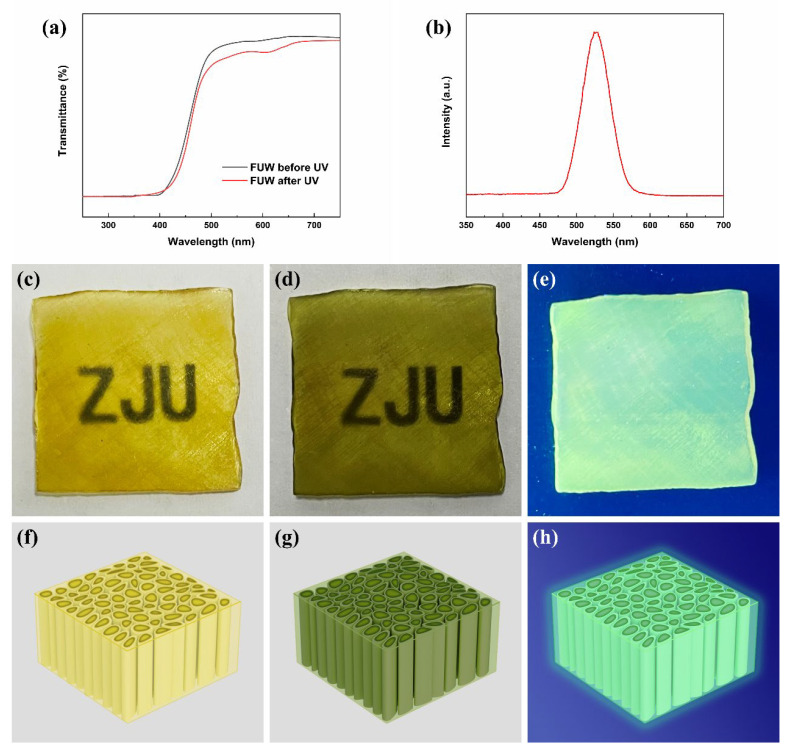
The UV–Vis spectra of FUW before and after exposure to UV (**a**); the fluorescence spectrum of FUW (**b**); the photos of FUW before the photochromic process (**c**), FUW after the photochromic process (**d**), and FUW under UV (**e**); the models of FUW before the photochromic process (**f**), FUW after the photochromic process (**g**), and FUW under UV (**h**).

**Figure 7:**
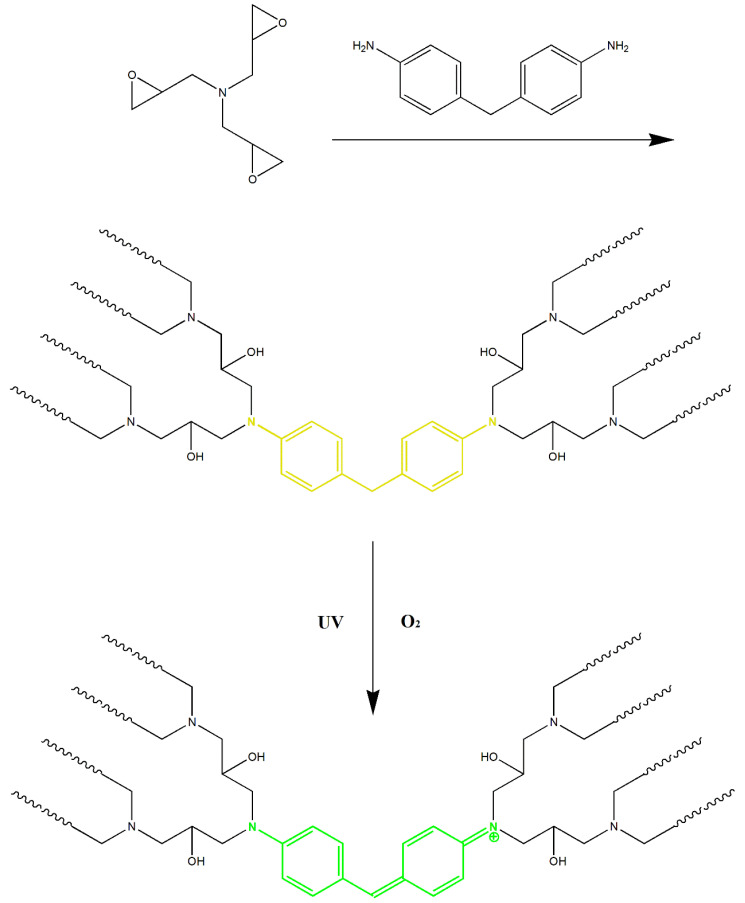
The change in the structure of tris(oxiran-2-ylmethyl)amine/DDM epoxy resin during the photochromic process.

The UV–Vis and fluorescence spectra of FUW are shown in Figure 6. Clearly, FUW was photo-responsive. The color of FUW before exposure to UV was yellow, which was caused by the oxidation of curing agent DDM. However, after exposure to UV, the color of FUW will change into dark green. This was due to the change in the molecular structure of epoxy resin under UV (Figure 7), which formed a large conjugated structure that can absorb red and yellow light (from 575 nm to 675 nm) [21]. It should be emphasized that the photo-response function of FUW was induced by the change in molecular structure of epoxy resin, rather than adding photo-responsive pigments [21]. Therefore, there was no escaping the problem of pigments, which may cause damage to the human body and pollute the environment.

Moreover, this large, conjugated structure will transform to the previous nonconjugated structure when heated, thereby changing the color of FUW back to yellow [21]. Under such circumstances, FUW was also thermal-responsive, and the photo-thermal-responsive stabilities are displayed in Figure S5. This reversible photo-thermal responsive FUW is expected to be used in the fields of anti-counterfeiting materials and sensor devices, which greatly broadens the application of wood.

Furthermore, this large, conjugated structure was also fluorescent, giving the fluorescence function to epoxy resin and FUW. Figure 6b showed that the dark green FUW can excite jade-green fluorescence (527 nm) under UV (260 nm). Similarly, the fluorescence function of FUW was also brought by the change of molecular structure, rather than the addition of fluorescent agents. In this case, there was no escaping the problem of fluorescent agents either, which may cause damage to the human body and pollute to environment. Combined with the good biocompatibility of wood, FUW with fluorescence function was expected to be used in the field of bioimaging, which further broadened the application of wood.

### 3.4. Water Resistance, Corrosion Resistance, and pH-Response of ORW and FUW

**Figure 8:**
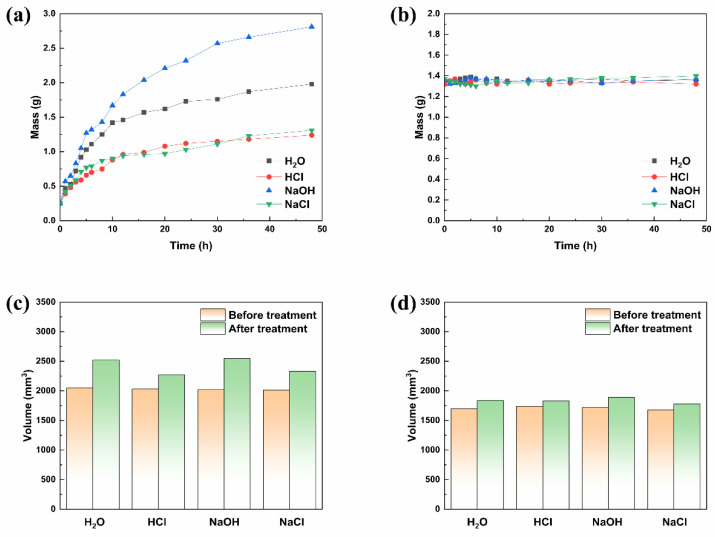
The mass change of ORW in H_2_O, HCl, NaOH, and NaCl (**a**); the mass change of FUW in H_2_O, HCl, NaOH, and NaCl (**b**); the volume change of ORW in H_2_O, HCl, NaOH, and NaCl (**c**); the volume change of FUW in H_2_O, HCl, NaOH, and NaCl (**d**).

One potential application of FUW is in smart windows. Window materials not only need high mechanical properties, but also the ability to resist chemical corrosion in extreme weather and environments, such as rain, acid rain, seawater and so on. Therefore, the water resistance and corrosion resistance of FUW and ORW were studied and discussed. Water resistance of ORW and FUW were displayed in Figure 8, Figures S6 and S7. Their swelling rates were recorded over time [31]. The results showed that the ORW absorbed water rapidly and expanded within 10 h; then, the swelling rate slowed down, and finally reached equilibrium at about 20 h. The ESR of ORW was 7.92. In contrast, the swelling rate of FUW was nearly 0 and the mass of FUW hardly changed after being soaked with water. The ESR of FUW was only 1.015. As a result, FUW had a higher water resistance than ORW, because the impregnated epoxy resin had good water resistance.

**Figure 9:**
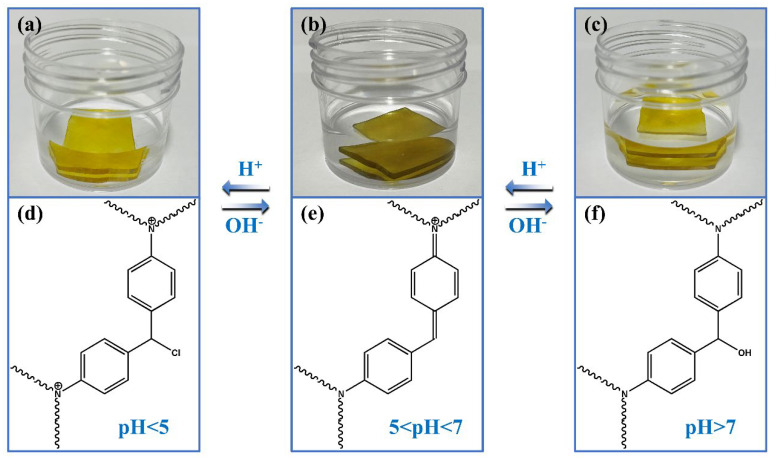
The pH−response of FUW: the photos of FUW in strong acid solution (**a**), weak acid solution or neutral solution (**b**), and alkali solution (**c**); the structures of FUW in strong acid solution (**d**), weak acid solution or neutral solution (**e**), and alkali solution (**f**).

Corrosion resistance of ORW and FUW was also illustrated in Figure 8, Figures S6 and S7. ORW and FUW were immersed in acid solution, alkali solution, and salt solution, respectively, and the changes in mass and volume were measured. Obviously, whether in acid solution, alkali solution, or salt solution, the mass and volume changes of FUW were smaller than ORW, indicating that FUW had better corrosion resistance than ORW. This was also because the impregnated epoxy resin had good corrosion resistance. Additionally, during the corrosion resistance test, we found that FUW also had a pH-response [32]. Under strong acidic and alkaline conditions, FUW appeared to be light yellow. Under neutral and weak acidic conditions, the color of FUW was dark green. The mechanism and structural changes are demonstrated in Figure 9.

## 4. Conclusions

Herein, we dipped tris (oxiran-2-ylmethyl)amine/DDM epoxy resin into delignified wood, and successfully synthesized a reversible photo-, thermal-, and pH-responsive FUW with fluorescence and high mechanical properties. The structure and morphology of ORW and FUW were characterized with SEM. The results showed that ORW was hollow whereas FUW was solid due to it filling with epoxy resin. This solid structure of FUW strongly enhanced the mechanical performance (tensile strength from 3.36 MPa to 28.54 MPa, elongation at break from 8.21% to 23.82%). At the same time, UV–Vis spectra revealed that, after exposure to UV, FUW could absorb orange light (from 575 nm to 675 nm) from the full spectrum of visible light. This was due to the change in the molecular structure of epoxy resin, and the formed large-conjugated structure can absorb orange light. Furthermore, the fluorescence spectrum showed that FUW also had fluorescence functions and will excite jade-green fluorescence (527 nm) under UV, which was excited from the large-conjugate structure of epoxy resin. Finally, water, salt, acid, and alkali tests revealed that FUW exhibited considerable water resistance (ESR was 1.015), corrosion resistance, and reversible pH responses, which was also due to the dipping of epoxy resin. This study provides ideas and methods for preparing novel multi-response FUW with high mechanical properties, which can be applied in fields of smart windows and sensors.

## Data Availability

Not applicable.

## Supplementary Materials

The following supporting information can be downloaded at: https://www.mdpi.com/article/10.3390/ma15031229/s1, Figure S1: The FTIR spectra of ORW, FUW and epoxy resin, Figure S2: The N_2_ adsorption-desorption isotherm of ORW, Figure S3: The N_2_ adsorption-desorption isotherm of delignified wood, Figure S4: The N_2_ adsorption-desorption isotherm of FUW, Figure S5: The photo of FUW before photochromic (a); the photo of FUW after photochromic (b); the UV–vis spectra of FUW at the first cycle and 100th cycle (c); the fluorescence spectra of FUW at the first cycle and 100th cycle(d), Figure S6: The volume change of ORW in H_2_O, HCl, NaOH and NaCl, Figure S7: The volume change of FUW in H_2_O, HCl, NaOH and NaCl; Table S1: The densities of FUW, ORW and epoxy resin.
