# Supplementary material for: Reversible Photo-, Thermal-, and pH-Responsive Functionalized Wood with Fluorescence Emission
Source: Materials (Basel). 2022 Feb 7;15(3):1229. doi: 10.3390/ma15031229 (PMC8840444; doi:10.3390/ma15031229)
Supplement: Supplementary file 1 [file materials-15-01229-s001.zip › materials-1533888-supplementary.pdf]

materials

Supplementary Materials

Reversible Photo-, Thermal-, and pH-Responsive Functionalized Wood with Fluorescence Emission

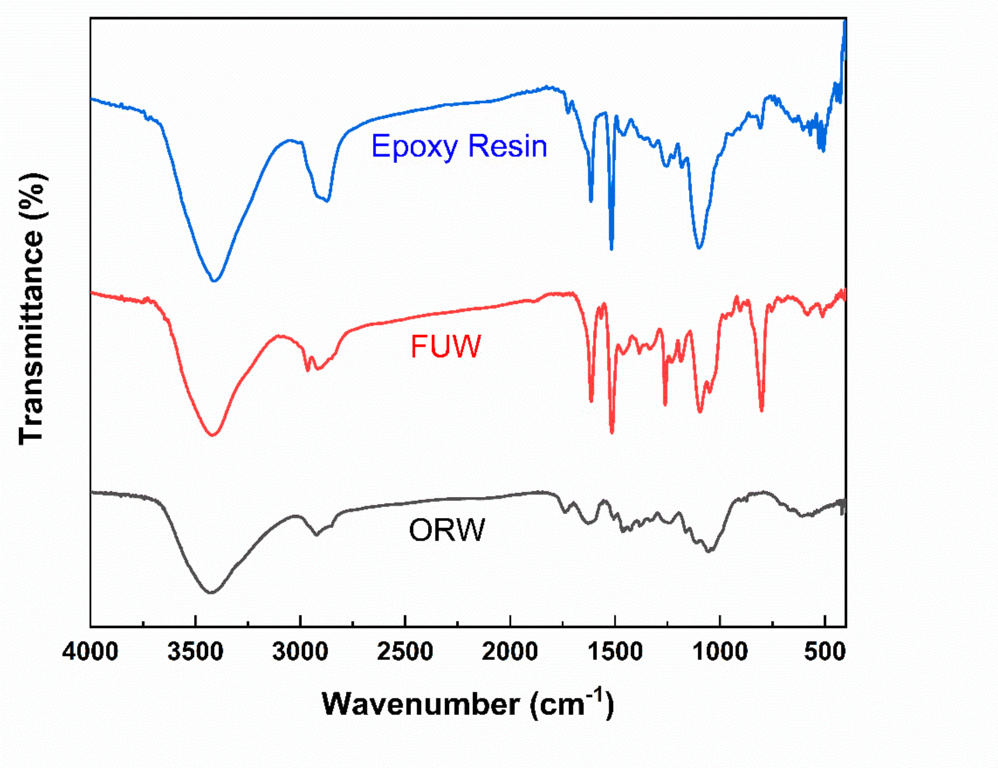

Figure S1. The FTIR spectra of ORW, FUW and epoxy resin.

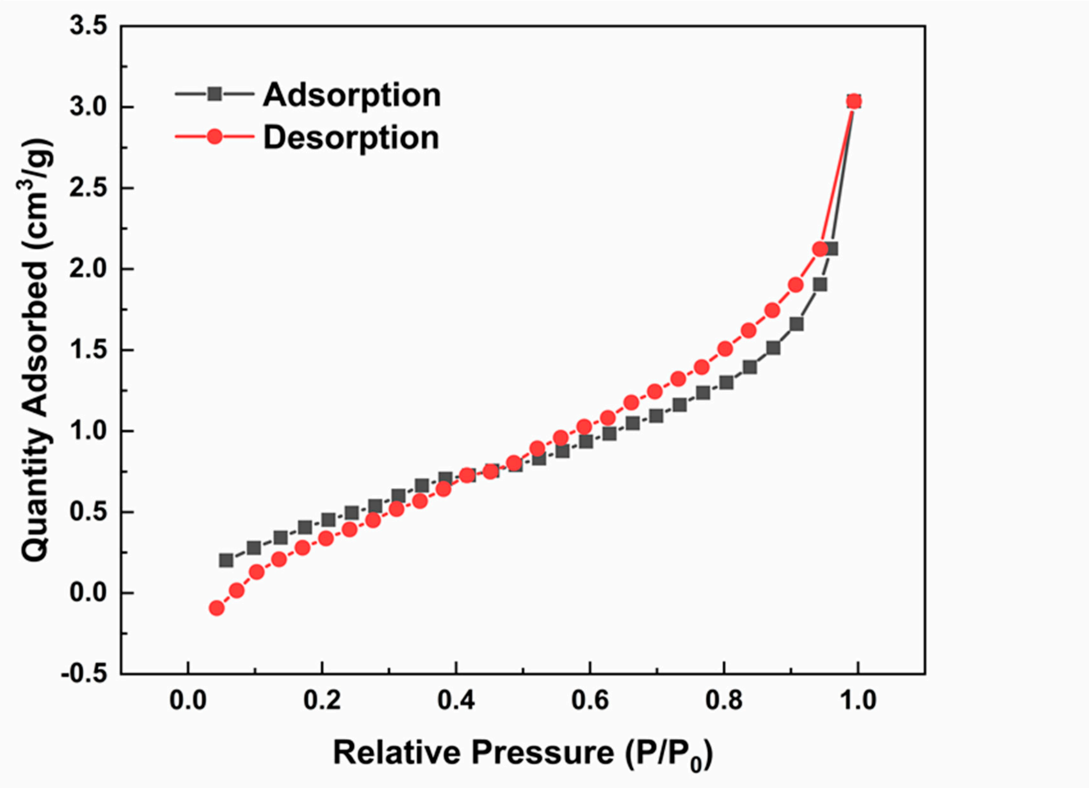

Figure S2. The N_2 adsorption-desorption isotherm of ORW.

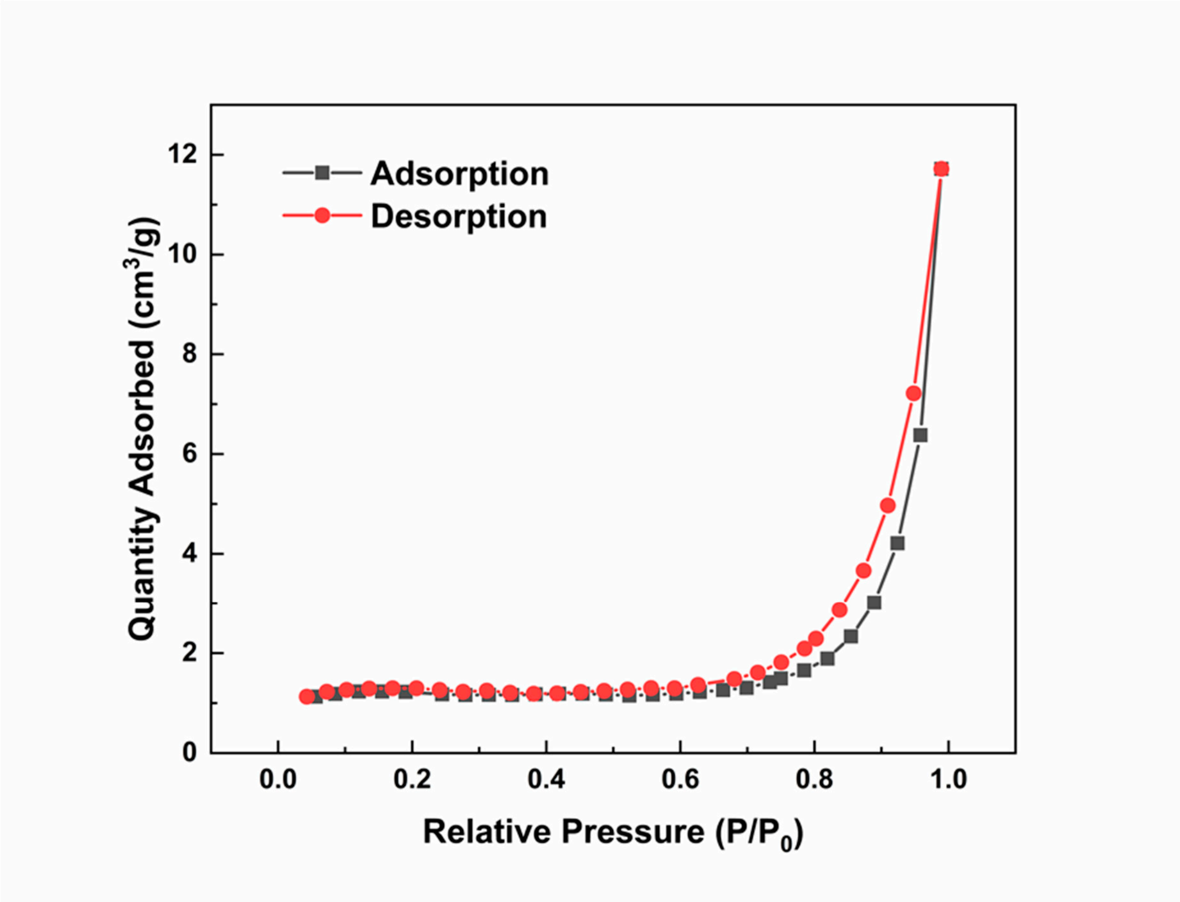

Figure S3. The N_2 adsorption-desorption isotherm of delignified wood.

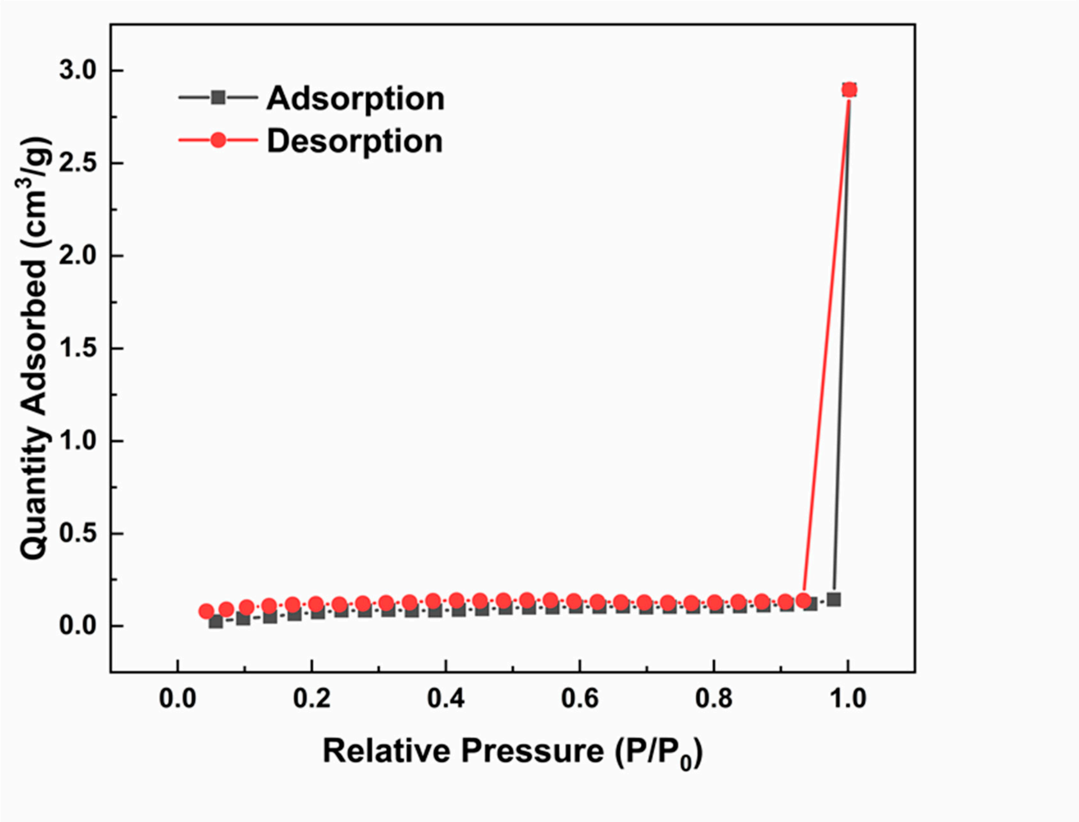

Figure S4. The N_2 adsorption-desorption isotherm of FUW.

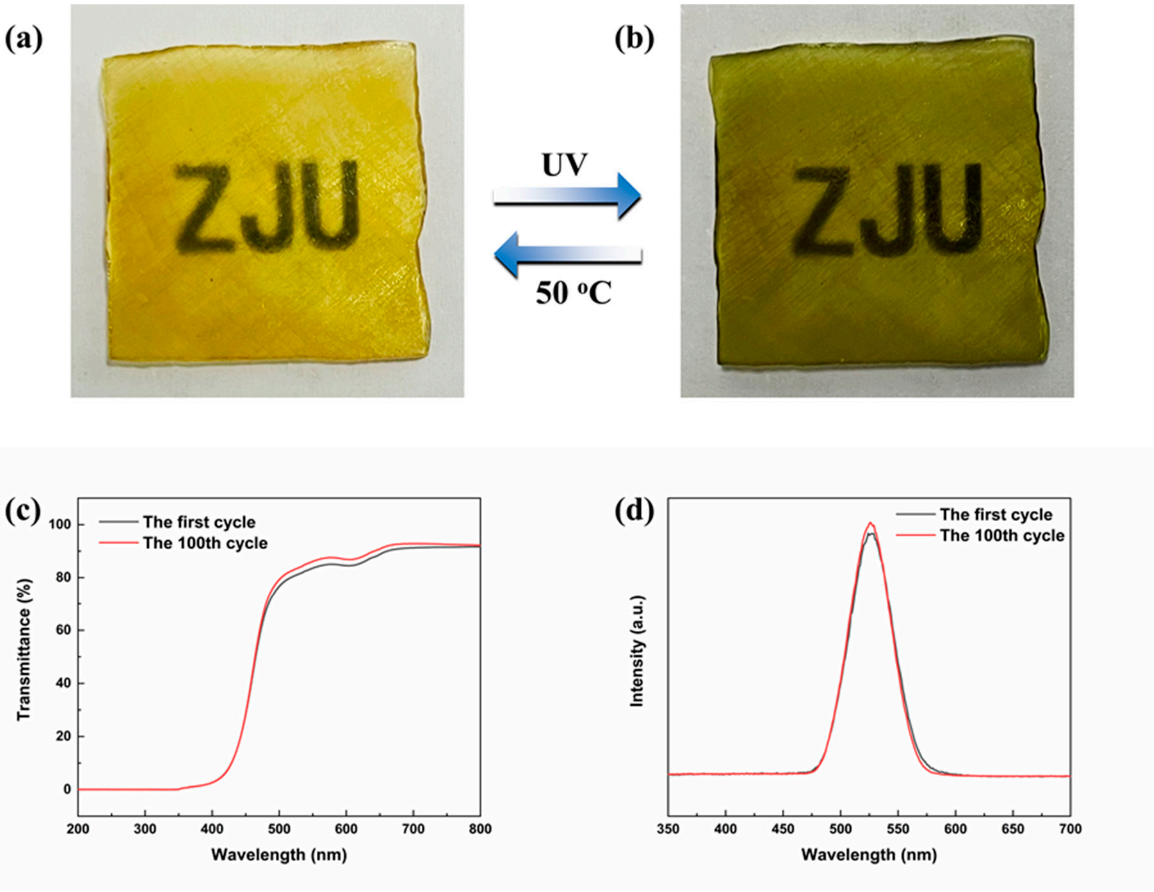

Figure S5. The photo of FUW before photochromic (**a**); the photo of FUW after photochromic (**b**); the UV-vis spectra of FUW at the first cycle and 100th cycle (**c**); the fluorescence spectra of FUW at the first cycle and 100th cycle(**d**).

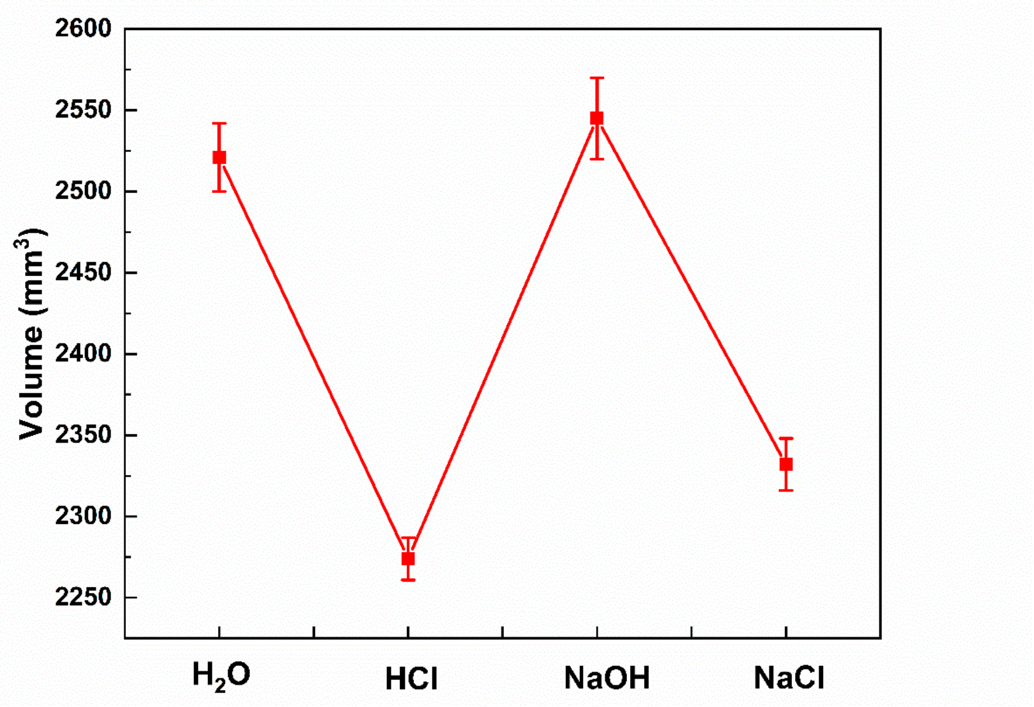

Figure S6. The volume change of ORW in H_2O, HCl, NaOH and NaCl.

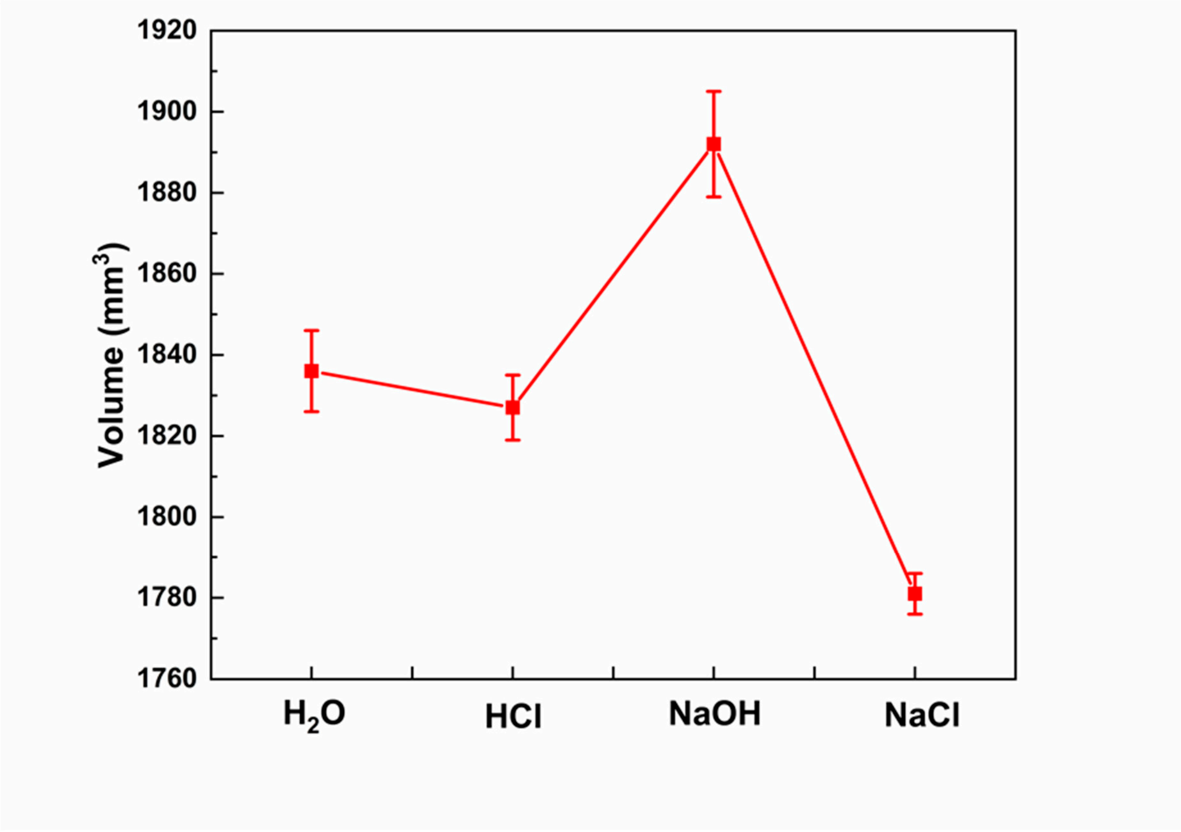

Figure S7. The volume change of FUW in H_2O, HCl, NaOH and NaCl.

Table S1. The densities of FUW, ORW and epoxy resin.

Samples	FUW	ORW	Epoxy Resin
**Density (g/cm^3)**	1.11	0.12	1.34
